# Melittin inhibits proliferation, migration and invasion of bladder cancer cells by regulating key genes based on bioinformatics and experimental assays

**DOI:** 10.1111/jcmm.14775

**Published:** 2019-11-05

**Authors:** Jie Yao, Zhan Zhang, Sheng Li, Bai Li, Xing‐Huan Wang

**Affiliations:** ^1^ Department of Urology Zhongnan Hospital of Wuhan University Wuhan China; ^2^ Department of Biological Repositories Zhongnan Hospital of Wuhan University Wuhan China; ^3^ Department of Rehabilitation Medicine Changhai Hospital Second Military Medical University Shanghai China

**Keywords:** antitumour, bioinformatics, bladder cancer, MAPK signalling pathway, melittin

## Abstract

The antitumour effect of melittin (MEL) has recently attracted considerable attention. Nonetheless, information regarding the functional role of MEL in bladder cancer (BC) is currently limited. Herein, we investigated the effect of MEL on critical module genes identified in BC. In total, 2015 and 4679 differentially expressed genes (DEGs) associated with BC were identified from the GSE31189 set and The Cancer Genome Atlas database, respectively. GSE‐identified DEGs were mapped and analysed using Gene Ontology and Kyoto Encyclopaedia of Genes and Genomes analyses to determine BC‐involved crucial genes and signal pathways. Coupled with protein–protein interaction network and Molecular Complex Detection analyses, Modules 2 and 4 were highlighted in the progression of BC. In in‐vitro experiments, MEL inhibited the proliferation, migration, and invasion of UM‐UC‐3 and 5637 cells. The expression of NRAS, PAK2, EGFR and PAK1 in Module 4—enriched in the MAPK signalling pathway—was significantly reduced after treatment with MEL at concentrations of 4 or 6 μg/mL. Finally, quantitative reverse transcription‐polymerase chain reaction and Western blotting analyses revealed MEL inhibited the expression of genes at the mRNA (ERK1/2, ERK5, JNK and MEK5), protein (ERK5, MEK5, JNK and ERK1/2) and phosphorylation (p‐ERK1/2, p‐JNK, and p‐38) levels. This novel evidence indicates MEL exerts effects on the ERK5‐MAK pathway—a branch of MAPK signalling pathway. Collectively, these findings provide a theoretical basis for MEL application in BC treatment.

## 1 INTRODUCTION

Bladder cancer (BC) is a malignant urological tumour associated with high incidence and mortality.^1^ Over the previous decades, the progress achieved in the management of BC has been modest and few new therapeutic agents against this disease were approved. However, fortunately, substantial improvement in our understanding of the biology linked to BC has taken place on the grounds of revolutionizing molecular analysis platforms and an increased interest in BC among physicians and scientists.^2^ These developments have advanced the investigation and discovery of promising novel therapeutic agents, targeted towards the prevention and reduction in recurrence and metastasis of BC.

Melittin (MEL) (C_131_H_229_N_39_O_31_; 2840 Da) is a major peptide constituent of bee venom, possessing the following properties 3, 4: (a) 40%–50% presence in the total dry weight of bee venom; (b) water solubility, linearity, cationicity, haemolysis and amphipathicity; (c) consists of 26 amino acids; (d) and contains a hydrophobic N‐terminal region with +4 charges and a hydrophilic C‐terminal region with +2 charges.

Melittin has been proposed as a promising agent for anticancer therapy. Reportedly, MEL is prominently involved in the regulation of multifarious cancers, exemplified by hepatocellular carcinoma,^5^, ^6^ breast cancer,^7^ lung cancer,^8^ leukaemia,^9^ ovarian carcinoma,^10^ gastric carcinoma,^11^ and prostate cancer.^12^ A handful of mechanisms of MEL cytotoxicity have been highlighted in various types of cancer cells (ie cell cycle alterations, effect on proliferation and/or inhibition of growth, induction of apoptosis.).^13^, ^14^ Nevertheless, research investigating the association between MEL and BC has been limited. In 1998, Winder et al^15^ demonstrated that MEL contributed to a complete loss of tumorigenicity in certain clones and reduced tumorigenicity in a human BC‐derived cell line. However, Jin et al^16^ presented evidence that MEL constrained the proliferative and migratory abilities of BC cells. The specific mechanism of MEL affecting BC and its potential use in anti‐BC therapy is worthy of investigation.

In recent years, microarrays employing high‐throughput platforms have emerged as a promising and effective approach for the detection of important genetic or epigenetic alterations in carcinogenesis and identification of promising biomarkers for the diagnosis and prognosis of cancers.^17^, ^18^ In fact, many gene expression profiles involved in various aspects cancers have been documented.^19^, ^20^, ^21^ However, additional critical genes and pathways associated with BC remain to be investigated.

**Figure 1:**
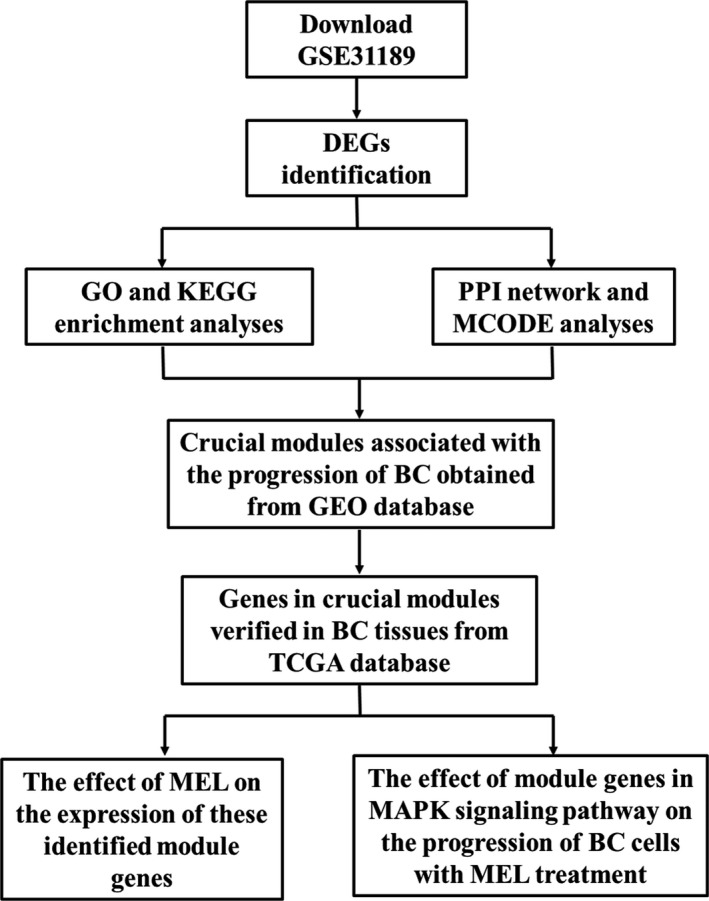
The pipeline designed for this work. DEGs, differentially expressed genes; GO, gene ontology; KEGG, Kyoto Encyclopaedia of Genes and Genomes; PPI, protein–protein interaction; MCODE, Molecular Complex Detection; BC, bladder cancer; GEO, Gene Expression Omnibus; TCGA, The Cancer Genome Atlas; MEL, melittin

The objective of the present study was to evaluate the effect of MEL on critical module genes identified in BC. Firstly, we independently used the Gene Expression Omnibus (GEO) and The Cancer Genome Atlas (TCGA) databases to identify BC‐associated differentially expressed genes (DEGs). Secondly, we detected the crucial genes and signalling pathways involved in BC through an analysis of GEO‐identified DEGs using the Gene Ontology (GO) and Kyoto Encyclopaedia of Genes and Genomes (KEGG) enriched signalling pathway. Subsequently, influential modules and their corresponding genes were determined through protein–protein interaction (PPI) network and Molecular Complex Detection (MCODE) analyses. Lastly, we analysed the expression of crucial module genes in BC tissues obtained from the TCGA database to investigate the effect of MEL and elucidate the molecular mechanism of MEL involved in the progression of BC. The design of the study is displayed in Figure 1.

## 2 MATERIALS AND METHODS

### 2.1 Microarray Data

The expression profile of GSE31189 was acquired from the GEO database (https://www.ncbi.nlm.nih.gov/geo/query/acc.cgi) based on the following two customized criteria: sample volume >30 and BC cells. The platform used for this analysis was the GPL570 (HG‐U133_Plus_2) Affymetrix Human Genome U133 Plus 2.0 Array. In total, 52 samples of human urothelial cancer cells and 40 of human non‐cancer urothelial cells were included. The data were obtained based on the overall methods developed by Urquidi et al^22^

The TCGA database catalogues major cancer‐causing genomic alterations with the purpose of establishing a comprehensive ‘atlas’ of cancer genomic profiles.^23^ Data (433 files) regarding BC were obtained based on criteria determined by HTSeq‐counts, transcriptome profiling and quantification of gene expression. In this analysis, 415 primary tumours and 19 normal solid tissues were included.

### 2.2 DEG screening

The R software (version 3.3.0; https://www.r-project.org/) was applied for data mining and analysis. In detail, the Limma package^24^ was available for DEG screening in the GEO data files with cut‐off criteria of absolute log_2_ fold change (FC) >1 and *P* < .05. The Deseq2 package^25^ in Bioconductor was applied for TCGA data files using the aforementioned criteria. False discovery rate analysis as the multiple testing was applied in the statistical analysis of DEGS screening.

### 2.3 Functional and pathway enrichment analyses

Featured by a community‐based bioinformatics resource, GO (http://www.geneontology.org) supplies information with respect to gene product function, adopting ontologies to convey biological knowledge.^26^ Similarly, KEGG (http://www.genome.jp/kegg/) is a bioinformatics resource including genomic, enzymatic pathways, chemicals and network information. It is useful in inspecting the functions and utilities of cells and organisms from both high‐level and genomic perspectives.^27^ GO enrichment and KEGG pathway analyses were performed to gain insight into the potential biological functions of DEGs in BC. The Database for Annotation, Visualization and Integrated Discovery (DAVID; http://david.ncifcrf.gov/) was applied for these analyses with a threshold of *P* < .05. Significantly enriched GO terms and KEGG pathways were screened using the *P* < .05 criterion and finally visualized through the R package ggplot2.^28^

### 2.4 Construction of the PPI network

The freely accessible, online, Search Tool for the Retrieval of Interacting Genes (STRING) database (http://string-db.org) includes established and predicted PPI.^29^ All DEGs were mapped onto the web‐based tool STRING to generate a PPI network, aiming to reveal the functions of proteins at the molecular level. Subsequently, the distribution characteristics of the DEGs in the PPI network were visualized using the Cytoscape software (http://www.cytoscape.org/). 30 The nodes and edges in the PPI network independently represented proteins and their interactions.

### 2.5 MCODE analysis

The MCODE (https://omictools.com/molecular-complex-detection-tool) utilizes a theoretical clustering algorithm for the detection of densely connected regions in large PPI networks, potentially representing molecular complexes.^31^ In this study, the MCODE was administered to mine the core protein complex in the constructed PPI network. Thereupon, significant module genes were annotated using the UniPro database (https://www.uniprot.org/).^32^

### 2.6 Cell culture and treatment

Human BC cell lines (UM‐UC‐3 and 5637) were obtained from the American Type Culture Collection. Cells were cultured in Dulbecco's modified Eagle's medium (Gibco BRL), supplemented with 10% foetal bovine serum (Gibco BRL) and penicillin/streptomycin at a concentration of 100 U/mL. The cultured cells were maintained at 37°C in a humidified atmosphere with 5% (v/v) CO_2_.

Melittin was purchased from Sigma–Aldrich Corp. BC cells were plated in 24‐well plates (5 × 10^4^ cells/well). Subconfluent cells were treated with MEL at various concentrations (0, 2, 4 and 6 μg/mL) for 24 hours.

### 2.7 Quantitative reverse transcription‐polymerase chain reaction (qRT‐PCR)

Total RNA was extracted from the UM‐UC‐3 and 5637 cell lines using the TRIzol reagent (Life Technologies) according to the instructions provided by the manufacturer. Total RNA (1 μg) was used as template to synthesize complementary DNA (cDNA) using a PrimeScript RT Reagent Kit with cDNA Eraser (Takara Biotechnology). Subsequently, qRT‐PCR was performed using the SYBR Premix Ex Taq (Takara Bio Inc). The primers utilized in this analysis are described in Table S1. All qRT‐PCR assays were performed on an ABI 7900 system (Applied Biosystems). β‐actin was used as an internal control to normalize the expression levels of genes. $2^{-\Delta \Delta C_{t}}$ method was applied to calculate the relative levels of gene expression.

### 2.8 Cell proliferation assay

The Cell Counting Kit‐8 (CCK‐8) assay (Dojindo Molecular Technologies, Inc) was used according to the protocol provided by the manufacturer to assess cell proliferation after treatment with MEL at various concentrations (ie 0, 2, 4 and 6 μg/mL). UM‐UC‐3 and 5637 cells were seeded in 96‐well plates and cultured at 37°C for 18 hours prior to treatment with MEL. After treatment (24 hours), the culture medium was replaced with Dulbecco's modified Eagle's medium containing 10% CCK‐8 solution. Each experiment was performed in triplicate. At 1‐4 days, the optical density (OD) was measured at a wavelength of 450 nm using a Multiskan FC (ThermoFisher Scientific, Inc).

### 2.9 Colony formation assay

Cells were seeded in 60‐mm plates (0.5 × 10^3^ cells/plate), cultured for 7 days, fixed with 10% formaldehyde for 5 minutes and stained with 1% crystal violet for 30 s prior to counting the number of colonies.

### 2.10 Cell migration assay

In the present study, both the scratch wound‐healing and transwell assays were employed to detect cell migration. The scratch wound‐healing assay was performed as previously reported.^16^ The initial gap length at 0 hours and residual gap length at 24 hours after wounding were calculated from photomicrographs. The transwell assay was performed in 24‐well Boyden chambers (Corning Inc) pre‐coated with the absence of Matrigel (BD Biosciences), as previously described.^33^ Cells were counted from at least four randomly selected microscopic fields.

### 2.11 Cell invasion assay

The in‐vitro invasion assay was performed in 24‐well Boyden chambers (Corning Inc) pre‐coated with the presence of Matrigel (BD Biosciences), as previously described.^33^ Cells were counted from at least four randomly selected microscopic fields.

### 2.12 Western blotting analysis

Protein extraction and Western blotting were conducted as previously reported.^33^ The following antibodies were used in the present study: JNK (Catalog: A11119; ABclonal Technology); p‐JNK (Catalog: AP0808; ABclonal Technology); MEK5 (Catalog: A6953; ABclonal Technology); p‐ERK1/2 (Catalog: AP0472; ABclonal Technology); ERK1/2 (Catalog: D160317; Sangon Biotech); p‐38 (Catalog: D155224; Sangon Biotech); p‐p38 (Catalog: D155179; Sangon Biotech); ERK5 (Catalog: D120601; Sangon Biotech); β‐Actin (Catalog: AC026; ABclonal Technology).

### 2.13 Statistical analysis

Data are expressed as mean ± standard deviation. Each experiment was performed in triplicate. The *t* test was applied to assess differences between two groups. One‐way and two‐way analysis of variance were employed to analyse multiple groups (>2) and two‐factor groups, respectively. A *P* < .05 denoted statistical significance. All statistical analyses were performed using the SPSS software (SPSS Inc).

## 3 RESULTS

### 3.1 Identification of DEGs in BC independently derived from the GEO and TCGA databases

**Figure 2:**
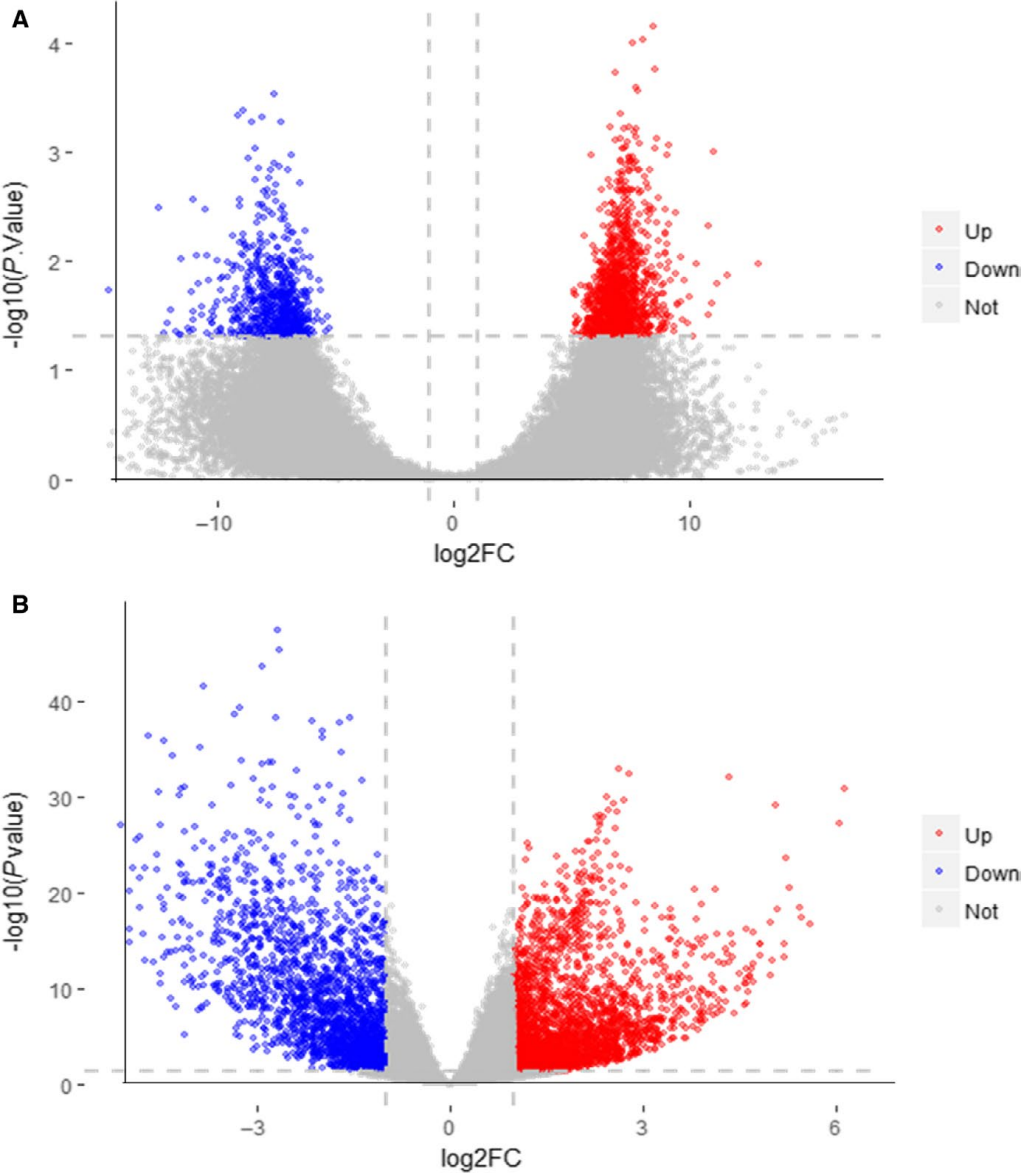
Volcano plot of detectable DEG profiles in BC. (A) Presentation of the volcano plot of DEGs identified from the GEO database; (B) Presentation of the volcano plot of DEGs identified from the TCGA. Red plots stand for up‐regulated genes, whereas blue plots indicate down‐regulated genes with the following criteria: *P* < .05 and absolute log_2_FC > 1. Grey plots indicate non‐significantly expressed genes. The abscissa presents the value of fold change in gene expression between BC group and control. The ordinate shows the − log10 of the adjusted *P* value for each gene, symbolizing the strength of the association. DEGs, differentially expressed genes; BC, bladder cancer; GEO, Gene Expression Omnibus; TCGA, The Cancer Genome Atlas; FC, fold change

Data related to BC were extracted from the GEO and TCGA databases for the identification of DEGs. Initially, one expression profile chip of BC—termed GSE31189—was used for the analysis, which stems from molecular markers for the detection of BC.^22^ According to the analysis performed using the Limma package (Figure 2A), a total of 2015 DEGs were identified in human urothelial cancer cells compared with non‐cancer urothelial cells. Among those, 1346 genes were up‐regulated, whereas 669 genes were down‐regulated. Transcriptomic data regarding BC—retrieved from the TCGA database—were analysed using the Deseq2 package^25^ to screen for DEGs. A total of 4679 DEGs were identified, of which 2644 and 2035 were up‐regulated and down‐regulated, respectively (Figure 2B).

### 3.2 GO Term and KEGG pathway enrichment analysis for GEO‐derived DEGs

**Figure 3:**
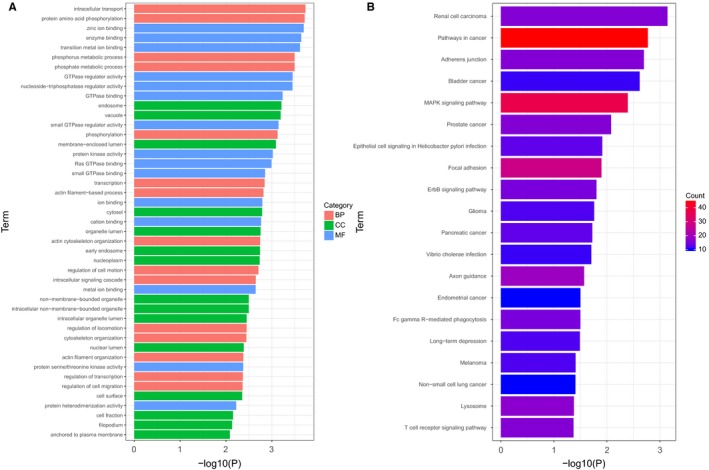
Top‐45 significant enriched GO terms of DEGs and top‐20 significant pathways associated with DEGs in BC according to the *P* value, determined through GO and KEGG analyses. GO, gene ontology; BP, biological process; MF, molecular function; CC, cellular component; DEGs, differentially expressed genes; BC, bladder cancer. KEGG, Kyoto Encyclopaedia of Genes and Genomes

Gene Ontology categories are classified into three groups, namely biological process (BP), cellular component (CC) and molecular function (MF). GO functional enrichment analysis of GEO‐derived DEGs was performed using the online biological tool DAVID with a threshold of *P* < .05. The top‐45 GO terms—according to the *P* value—were determined (Figure 3A). In the case of BP, GO terms mainly included intracellular transport, protein amino acid phosphorylation, phosphate metabolic process, phosphorylation, transcription, actin filament‐based process, regulation of cell motion. These terms play crucial roles in the progression of cancer. For the CC, the GO terms principally included endosome, vacuole, membrane‐enclosed lumen, cytosol, early endosome, nucleoplasm, non‐membrane bounded organelle, intracellular non‐membrane bounded organelle, intracellular organelle lumen. For the MF, the GO terms predominantly included plasma membrane, zinc ion binding, enzyme binding, transition metal ion binding, GTPase regulator activity, nucleoside‐triphosphatase regulator activity, GTPase binding, small GTPase regulator activity. The results demonstrated that these identified DEGs associated with BC are involved in the progression of tumours of all types.

Subsequently, KEGG pathway analysis was performed to further evaluate the biological significance of the DEGs using *P* < .05 as a criterion. As presented in Figure 3B and Table 1, the top‐20 significant pathways relevant to the DEGs were identified and visualized using the R package ggplot2. These included pathways in cancer, the MAPK signalling pathway, focal adhesion, the ErbB signalling pathway and BC. This evidence further indicated that these identified DEGs associated with BC may be involved in the progression of cancer. Furthermore, the DEGs likely encompass key molecules associated with the development or progression of tumours.

**Table 1:** The module genes in BC were involved in the top‐20 significant signalling pathways ranked according to *P* value

Term	Count	DEGs
Renal cell carcinoma	16	TGFB3, HGF, CDC42, MAPK1, **NRAS**, **PAK2**, ETS1, ARAF, VEGFA, GAB1, CDC42P2, TCEB2, **PAK1**, TCEB1, AKT3, AKT2, FH
Pathways in cancer	45	FGFR2, WNT5A, E2F2, PDGFA, MMP9, ERBB2, STK36, MITF, PML, TGFB3, MMP1, SHH, FLT3LG, CDC42, IGF1R, ACVR1B, BCL2, CDC42P2, HHIP, FAS, WNT6, AXIN2, TPR, TRAF5, CSF2RA, AKT3, AKT2, FH, **EGFR**, COL4A2, AR, IL8, TP53, HGF, RALGDS, CTNNA3, PRKCB, MAPK1, **NRAS**, CCDC6, LAMA4, ETS1, ARAF, VEGFA, TCEB2, TCEB1
Adherens junction	16	**EGFR**, ACTN4, ERBB2, CTNND1, SNAI2, CSNK2A1P, SNAI1, CTNNA3, FARP2, IGF1R, CDC42, ACVR1B, MAPK1, CSNK2A1, SORBS1, **FYN**, CDC42P2, WASL
Bladder cancer	11	**EGFR**, E2F2, MAPK1, **NRAS**, IL8, MMP9, ERBB2, ARAF, VEGFA, TP53, MMP1
MAPK signalling pathway	37	FGFR2, PDGFA, MRAS, TGFB3, CDC42, ACVR1B, MAP3K3, **PAK2**, RASGRP1, DUSP16, CDC42P2, HSPA6, HSPA7, PPP3CA, **PAK1**, PRKACB, FAS, NFATC2, AKT3, AKT2, **EGFR**, TAOK1, CACNA1I, TP53, TAB1, STK3, FLNA, PRKCB, MAPK1, **NRAS**, MAP4K4, RASGRF2, RPS6KA2, RRAS2, CACNA1E, GADD45B, PLA2G5, DUSP6, MAP3K11
Prostate cancer	16	**EGFR**, FGFR2, E2F2, AR, PDGFA, CREB1, ERBB2, TP53, NRAS, IGF1R, MAPK1, BCL2, ARAF, SRD5A2, AKT3, AKT2
L2,Epithelial cell signalling in Helicobacter pylori infection	13	**EGFR**, IL8, CCL5, **ATP6V0B**, **ATP6V1F**, **ATP6V1C1**, **ATP6V0C**, CDC42, **ATP6V1E2**, CDC42P2, HBEGF, **PAK1**, JAM3, **ATP6V0A2**
Focal adhesion	28	CAV3, PDGFA, ERBB2, ITGB5, CDC42, IGF1R, DOCK1, **PAK2**, ITGB8, BCL2, CDC42P2, **PAK1**, AKT3, AKT2, **EGFR**, COL4A2, VAV3, ACTN4, ROCK1, MYLK3, HGF, FLNA, PRKCB, MAPK1, LAMA4, ROCK1P1, **FYN**, VEGFA, RELN, PARVA
ErbB signalling pathway	15	**EGFR**, NRG4, ERBB4, ERBB2, CAMK2G, PRKCB, **NRAS**, MAPK1, **PAK2**, ARAF, GAB1, HBEGF, **PAK1**, AKT3, AKT2
Glioma	12	**EGFR**, E2F2, IGF1R, MAPK1, NRAS, PDGFA, CAMK2G, ARAF, TP53, AKT3, PRKCB, AKT2
Pancreatic cancer	13	**EGFR**, E2F2, ERBB2, TP53, TGFB3, RALGDS, ACVR1B, MAPK1, CDC42, ARAF, VEGFA, CDC42P2, AKT3, AKT2
Vibrio cholerae infection	11	**ATP6V0C**, **ATP6V1C1**, ADCY9, **ATP6V1E2**, CFTR, PRKACB, KDELR1, **ATP6V0A2**, **ATP6V0B**, PRKCB, **ATP6V1F**
Axon guidance	19	GNAI3, ROCK1, SLIT2, **EPHB1**, **EPHB2**, SEMA5A, CDC42, MAPK1, SEMA6A, **NRAS**, **PAK2**, ROCK1P1, UNC5A, **FYN**, CFL2, CDC42P2, EFNA5, PPP3CA, **PAK1**, NFATC2, SRGAP2
Endometrial cancer	10	**EGFR**, MAPK1, **NRAS**, ERBB2, ARAF, TP53, AXIN2, AKT3, CTNNA3, AKT2
Fc gamma R‐mediated phagocytosis	15	VAV3, ASAP1, ASAP3, PRKCB, CDC42, MAPK1, GSN, CFL2, SCIN, CDC42P2, FCGR2A, **PAK1**, INPP5D, WASL, AKT3, AKT2
Long‐term depression	12	IGF1R, MAPK1, **NRAS**, NOS1, GNAI3, GNAQ, GNA11, ARAF, CRH, GRID2, PLA2G5, PRKCB
Melanoma	12	**EGFR**, E2F2, IGF1R, MAPK1, **NRAS**, PDGFA, ARAF, MITF, TP53, HGF, AKT3, AKT2
Non‐small cell lung cancer	10	**EGFR**, E2F2, MAPK1, **NRAS**, ERBB2, ARAF, TP53, AKT3, PRKCB, AKT2
Lysosome	17	ARSB, ABCB9, LIPA, ACP5, **ATP6V0B**, MANBA, CTSW, AP1S3, **ATP6V0C**, NPC1, AP1S2, IDS, GLA, CTSB, CTNS, CLN5, **ATP6V0A2**
T cell receptor signalling pathway	16	PDK1, VAV3, CD8B, CDC42, CARD11, MAPK1, **NRAS**, **PAK2**, **FYN**, RASGRP1, CDC42P2, ZAP70, **PAK1**, PPP3CA, NFATC2, AKT3, AKT2

Note

The bold presents the key genes in following Module 2 and 4.

Abbreviations: DEGs, differentially expressed genes; KEGG, the Kyoto Encyclopaedia of Genes and Genomes.

### 3.3 Construction of the PPI network and MCODE analyses

**Figure 4:**
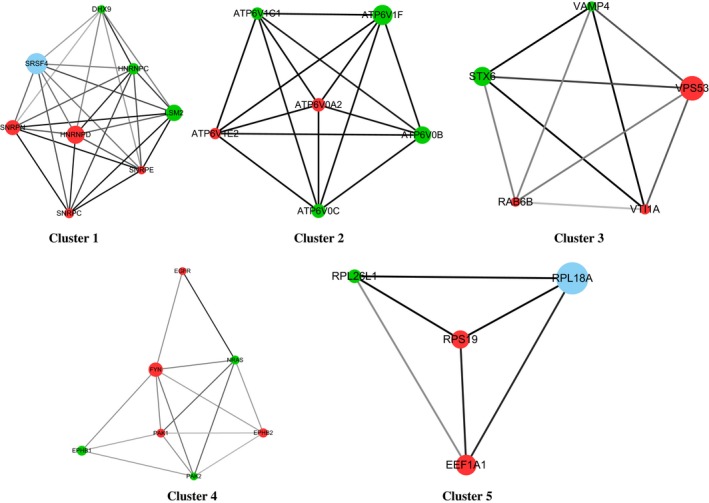
Five module networks were analysed using the MCODE algorithm (https://omictools.com/molecular-complex-detection-tool). The red nodes stand for up‐regulated genes, whereas the green nodes indicate down‐regulated genes. The blue nodes indicate genes that are not differentially expressed, but interact with DEGs in BC. The diameter of the node exhibited a negative relationship with the *P* value, indicating the significance of the node. The line between two nodes shows the interaction between two genes. The depth of the line indicates the strength of their interaction. MCODE, Molecular Complex Detection; BC, bladder cancer; DEGs, differentially expressed genes

The GEO‐identified DEGs were mapped onto the STRING database to assess their interactive relationship, visualized through the Cytoscape software and analysed using the MCODE algorithm (a plug‐in of Cytoscape). Subsequently, the PPI network was constructed using the criterion of interacting pairs with a combined score >0.9 and visualized through the Cytoscape software (Figure S1). The five modules listed in Table 2 were generated using the custom parameters of 0.2 Node Score Cut‐off and 3K‐Core, analysed through the MCODE. The visualized results are presented in Figure 4. Combined with the results of the KEGG enrichment analysis, it was revealed that Modules 2 and 4 may be involved in the progression of BC. The Module 2 and 4 genes enriched in the significant signalling pathways are marked in red in Table 1. Notably, Module 4 was comprised of EPHB2, FYN, NRAS, PAK2, EPHB1, EGFR and PAK1. Among these, NRAS, PAK2, EGFR and PAK1 were enriched in the MAPK signalling pathway. Module 2 included six genes (ie ATP6V0B, ATP6V1C1, ATP6V1E2, ATP6V0C, ATP6V1F and ATP6V0A2). Using the UniPro database,^32^ these genes were shown to partly constitute the V‐ATPase domain (Table 3). Collectively, the crucial genes in Modules 2 and 4 were shown to be involved in the progression of BC, while the gens NRAS, PAK2, EGFR and PAK1 in Module 4 were enriched in the MAPK signalling pathway.

**Table 2:** Five modules from the PPI networks analysed using the MCODE algorithm

Cluster	Score	Nodes	Edges	Node IDs
1	7.714	8	27	LSM2, SNRPE, DHX9, HNRNPC, SRSF4, SNRPN, HNRNPD, SNRPC
2	6	6	15	ATP6V0B*, ATP6V1C1*, ATP6V1E2*, ATP6V0C*, ATP6V1F*, ATP6V0A2*
3	5	5	10	STX6, VAMP4, VTI1A, VPS53, RAB6B
4	5	7	15	EPHB2*, FYN*, **NRAS** *,#, **PAK2** *,#, EPHB1^*^, **EGFR** *,#, **PAK1** *,#
5	4	4	6	RPL18A, RPL26L1, RPS19, EEF1A1

Note

The red stand for the module genes enriched in significant signalling pathways and the red in bold represent genes in each module enriched in MAPK signalling pathway.

Abbreviations: MCODE, Molecular Complex Detection; PPI, protein‐protein interaction.

^*^ Stand for the module genes enriched in significant signaling pathways.

^#^ represent genes in each module enriched in MAPK signaling pathway.

**Table 3:** Annotation of DEGs in Module 2 according to the UniPro database

Domain	Gene	Synonyms	Protein	Function
V0	ATP6V0B	ATP6F	V‐type proton ATPase 21 kD proteolipid subunit	Proton‐conducting pore forming subunit of the membrane integral V0 complex of vacuolar ATPase.
	ATP6V0C	ATP6C, ATP6L, ATPL	V‐type proton ATPase 16 kD proteolipid subunit	Proton‐conducting pore forming subunit of the membrane integral V0 complex of vacuolar ATPase.
	ATP6V0A2	/	V‐type proton ATPase 116 kD subunit a isoform 2	Part of the proton channel of V‐ATPases. Essential component of the endosomal pH‐sensing machinery.
V1	ATP6V1C1	ATP6C, ATP6D, VATC	V‐type proton ATPase subunit C 1	Subunit of the peripheral V1 complex of vacuolar ATPase. Subunit C is necessary for the assembly of the catalytic sector of the enzyme and is likely to have a specific function in its catalytic.
	ATP6V1E2	ATP6E1, ATP6EL2, ATP6V1EL2	V‐type proton ATPase subunit E 2	Subunit of the peripheral V1 complex of vacuolar ATPase essential for assembly or catalytic function. This isoform is essential for energy coupling involved in acidification of acrosome.
	ATP6V1F	ATP6S14, VATF	V‐type proton ATPase subunit F	Subunit of the peripheral V1 complex of vacuolar ATPase essential for assembly or catalytic function.

Abbreviation: DEGs, differentially expressed genes.

### 3.4 Verification of the pivotal module genes identified from the GSE31189 set in BC tissues from the TCGA database

We generated the overlap of DEGs independently identified from the GEO and TCGA databases to further identify the expression of crucial module genes in BC tissues. These two databases had 246 DEGs in common (Figure S2). In addition, the majority of crucial genes in Modules 2 and 4—apart from ATP6V1E2, ATP6V0C, ATP6V0A2 and PAK1—exhibited similar differential expression in BC tissues obtained from the TCGA database, as listed in Table 4. Hence, most pivotal module genes associated with the progression of BC were also verified in BC tissues from TCGA database.

**Table 4:** Genes in Modules 2 and 4 differentially expressed in the GEO and TCGA databases

Symbol	GEO		Symbol	TCGA	
	Log_2_FC	*P* value		Log_2_FC	*P* value
EPHB1	−6.972	2.506E‐02	EPHB1	−2.831	8.880E‐13
ATP6V0B	−9.094	2.960E‐02	ATP6V0B	−0.8616	1.620E‐09
FYN	8.089	4.733E‐02	FYN	1.446	1.350E‐08
EPHB2	6.227	2.178E‐02	EPHB2	1.624	5.050E‐08
ATP6V1F	−8.246	3.815E‐02	ATP6V1F	−0.5494	1.190E‐05
NRAS	−7.073	2.040E‐02	NRAS	−0.6108	2.510E‐05
ATP6V1C1	−8.096	1.377E‐02	ATP6V1C1	−0.4632	6.980E‐04
PAK2	−7.894	1.453E‐02	PAK2	−0.2310	1.863E‐02
EGFR	8.224	1.456E‐02	EGFR	0.6987	4.591E‐02
ATP6V1E2	7.139	1.062E‐02	ATP6V1E2^a^	0.3310	5.472E‐02
ATP6V0C	−6.727	1.881E‐02	ATP6V0C^a^	0.1403	0.3170
ATP6V0A2	6.940	1.716E‐02	ATP6V0A2^a^	0.07614	0.4683
PAK1	7.556	1.970E‐02	PAK1^a^	−0.1279	0.4696

Abbreviations: FC, fold change; GEO, Gene Expression Omnibus; TCGA, The Cancer Genome Atlas.

a Non‐significant differentially expressed genes.

### 3.5 MEL inhibits cell proliferation, migration and invasion in UM‐UC‐3 and 5637 cells

Prior to functional experiments, the half maximal inhibitory concentration (IC_50_) of MEL in BC cells was evaluated. We analysed the inhibitory rate after treatment with MEL at various concentrations (ie 1, 3, 5, 7, 9 and 12 μg/mL) for 24 hours in UM‐UC‐3 and 5637 cells. Results revealed that MEL constrained the cell proliferation of BC in a concentration‐dependent manner (Figure S3). At 24 hours after treatment, the IC_50_ of MEL for the inhibition of cell growth was 7.6 and 8.2 μg/mL in UM‐UC‐3 and 5637 cells, respectively. Therefore, MEL at concentrations of 0, 2, 4 and 6 μg/mL was used for the following experiments.

**Figure 5:**
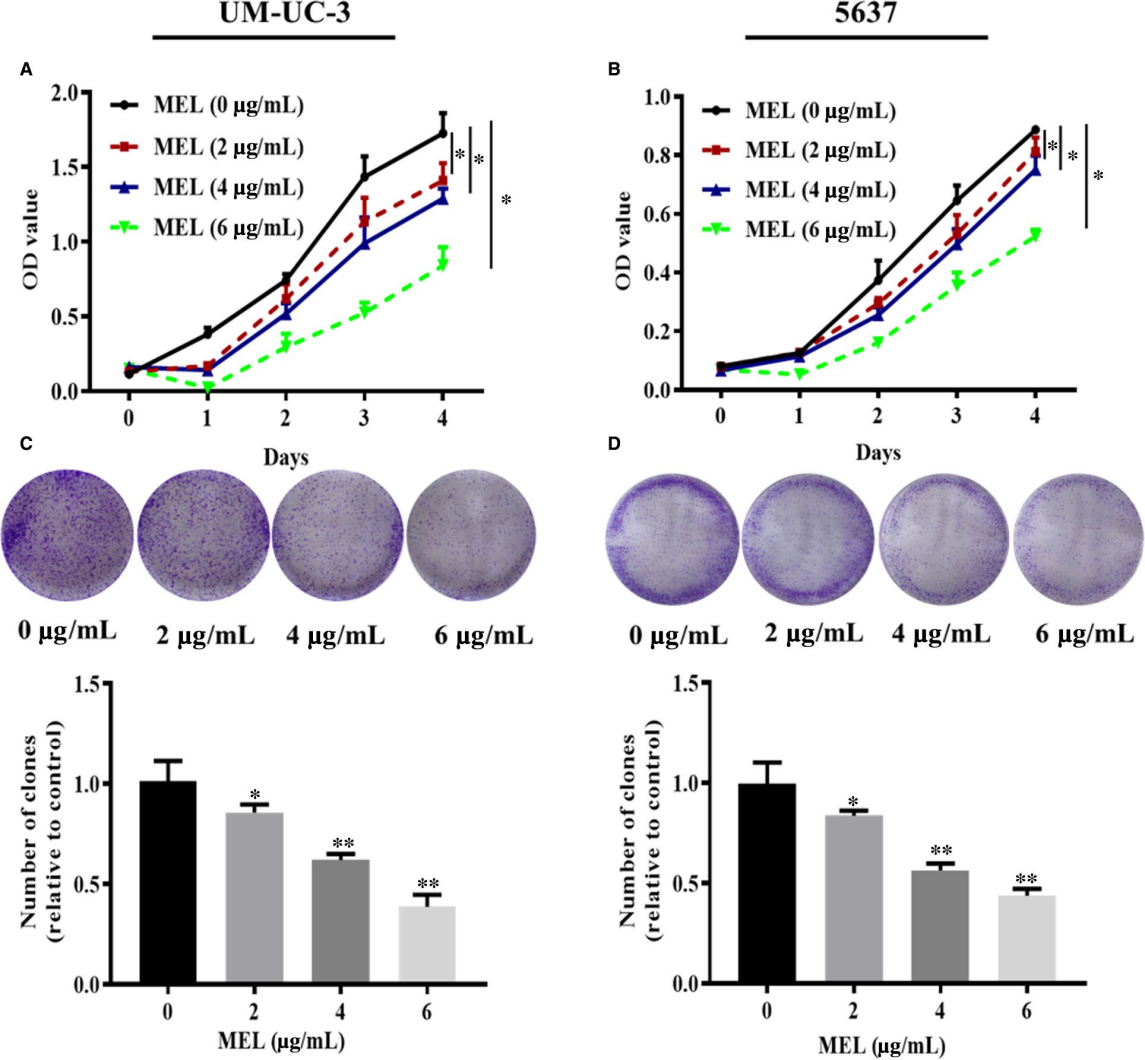
MEL inhibited cell proliferation in UM‐UC‐3 and 5637 cells. The proliferation of (A) UM‐UC‐3 and (B) 5637 cells was detected through CCK‐8 assay after treatment with MEL (0, 2, 4, or 6 μg/mL). *, *P* < .05. The proliferation of (C) UM‐UC‐3 and (D) 5637 cells was detected by colony formation assay after treatment with MEL (0, 2, 4, or 6 μg/mL). *, *P* < .05; **, *P* < .01. MEL, melittin; CCK‐8, Cell Counting Kit*‐*8

**Figure 6:**
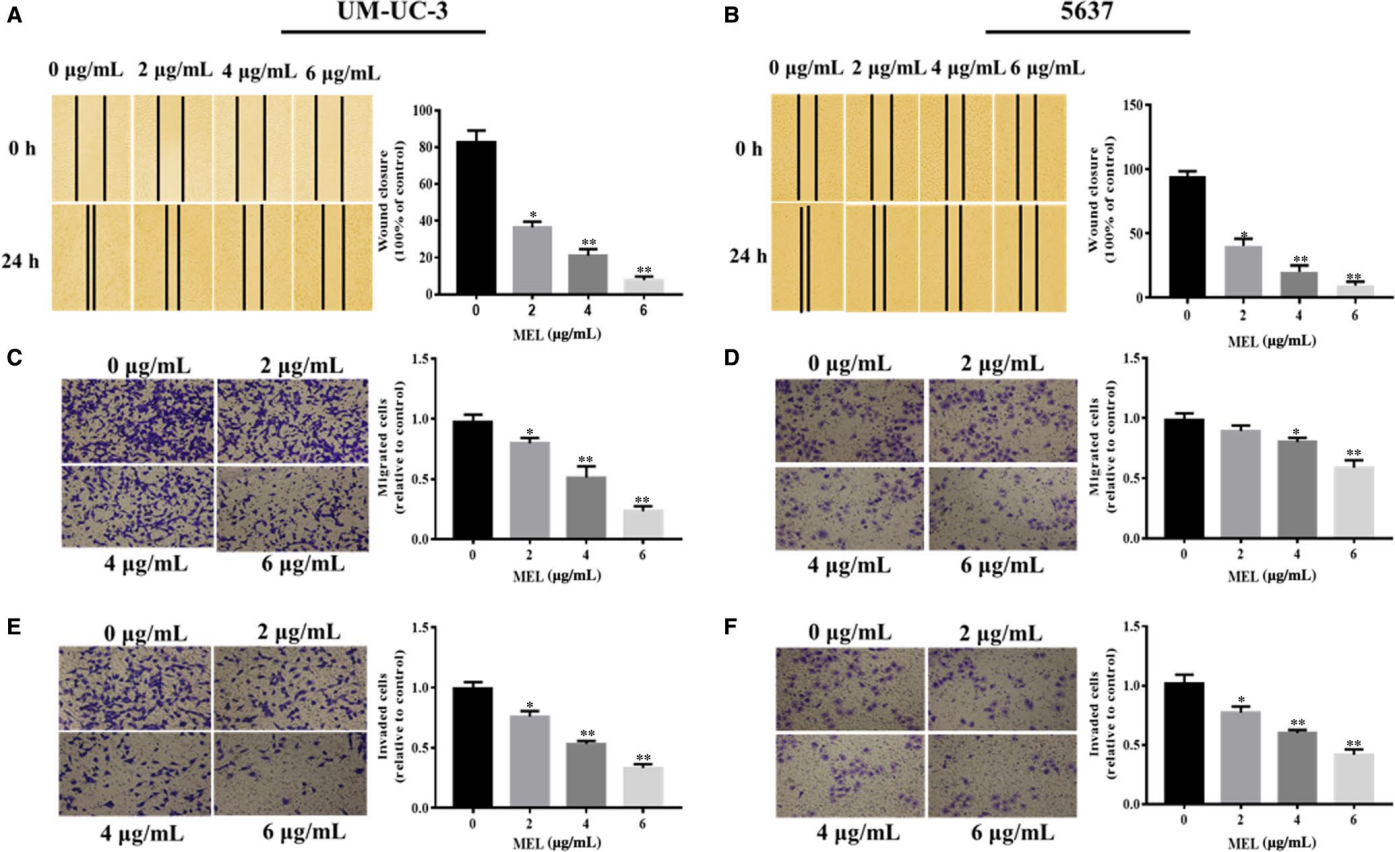
MEL inhibited cell migration, and invasion in UM‐UC‐3 and 5637 cells. The migration of (A) UM‐UC‐3 and (B) 5637 cells was detected through the scratch wound‐healing assay after treatment with MEL (0, 2, 4, or 6 μg/mL). *, *P* < .05; **, *P* < .01. The migration of (C) UM‐UC‐3 and (D) 5637 cells was detected through the transwell assay after treatment with MEL (0, 2, 4, or 6 μg/mL). *, *P* < .05; **, *P* < .01. The invasion of (E) UM‐UC‐3 and (F) 5637 cells was detected through the transwell assay after treatment with MEL (0, 2, 4, or 6 μg/mL). *, *P* < .05; **, *P* < .01. MEL, melittin

Previous studies noted the important roles of MEL in the progression of various cancer cells.^13^, ^14^ Therefore, we investigated the effect of MEL on the proliferation, migration and invasion of UM‐UC‐3 and 5637 cells through CCK‐8, colony formation, scratch wound‐healing and transwell assays. The results of the CCK‐8 and colony formation assays showed a decreasing trend in cell proliferation in the MEL‐treated group (2, 4 or 6 μg/mL) compared with the control (Figure 5A‐D, *P* < .05, *P* < .01). Similar results were obtained regarding the effect of MEL on cell migration, analysed using the scratch wound‐healing (Figure 6A,B, *P* < .05, *P* < .01) and transwell assays (Figure 6C,D, *P* < .05, *P* < .01). In addition, the transwell assay revealed a reducing trend in cell invasion in the MEL‐treated group (2, 4, or 6 μg/mL) compared with the control (Figure 6E,F, *P* < .05, *P* < .01). Collectively, these results demonstrated that MEL inhibited cell proliferation, migration and invasion in UM‐UC‐3 and 5637 cells.

### 3.6 MEL constrains the expression of pivotal module genes in UM‐UC‐3 and 5637 cells

**Figure 7:**
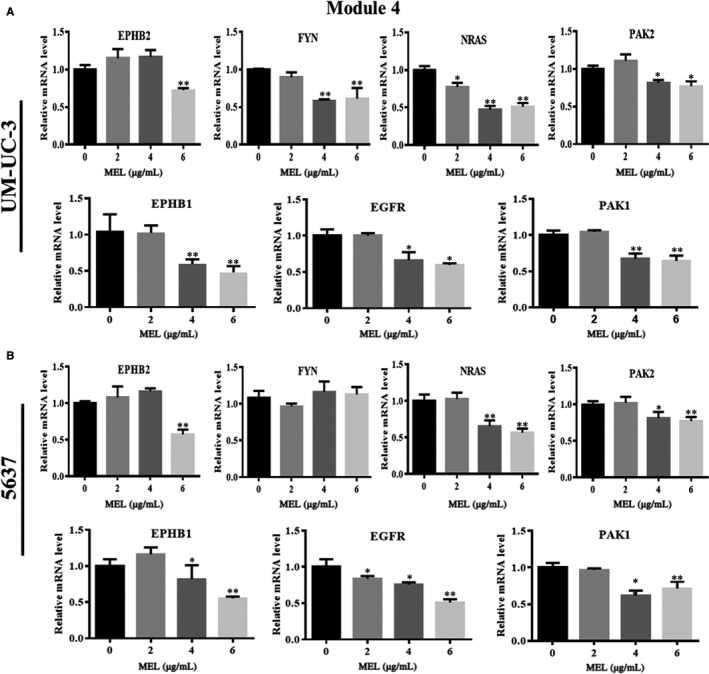
MEL inhibited the expression of pivotal genes in Module 4, as shown by qRT‐PCR in UM‐UC‐3 and 5637 cells. The mRNA expression of seven genes in Module 4 (ie EPHB2, FYN, NRAS, PAK2, EPHB1, EGFR, and PAK1) was determined through qRT‐PCR in (A) UM‐UC‐3 and (B) 5637 cells after treatment with MEL (0, 2, 4, or 6 μg/mL); *, *P* < .05; **, *P* < .01. MEL, melittin; qRT‐PCR, quantitative reverse transcription‐polymerase chain reaction

**Figure 8:**
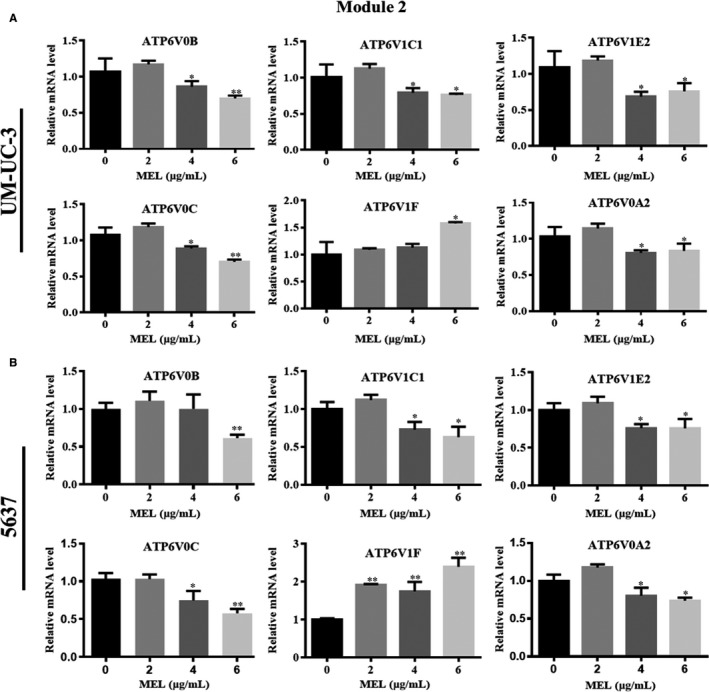
MEL inhibited the expression of pivotal genes in Module 2, as shown by qRT‐PCR in UM‐UC‐3 and 5637 cells. The mRNA expression of six genes in Module 2 (ie ATP6V0B, ATP6V1C1, ATP6V1E2, ATP6V0C, ATP6V1F, and ATP6V0A2) was determined through qRT‐PCR in (A) UM‐UC‐3 and (B) 5637 cells after treatment with MEL (0, 2, 4, or 6 μg/mL); *, *P* < .05; **, *P* < .01. MEL, melittin; qRT‐PCR, quantitative reverse transcription‐polymerase chain reaction

We investigated the effect of MEL on the expression of pivotal module genes (Modules 4 and 2) identified in UM‐UC‐3 and 5637 cells. The genes in Module 4 (ie EPHB2, FYN, NRAS, PAK2, EPHB1, EGFR, and PAK1) were down‐regulated in the MEL‐treated group (6 μg/mL) compared with the control (0 μg/mL; Figure 7A,B, *P* < .05, *P* < .01). There was no significant difference found in the expression of most genes in Module 4 between the MEL‐treated group (2 μg/mL) and the control (0 μg/mL; Figure 7A,B). In the aforementioned results, we revealed that NRAS, PAK2, EGFR and PAK1 were associated with the MAPK signalling pathway (Tables 1 and 2). These four genes were significantly affected by treatment with MEL at a concentration of 4 or 6 μg/mL in both cell lines (Figure 7A,B, *P* < .05, *P* < .01). Similarly, the genes in Module 2 associated with V‐ATPase—apart from ATP6V1F—were down‐regulated in the MEL‐treated group (4 or 6 μg/mL) compared with the control (0 μg/mL; Figure 8A,B, *P* < .05, *P* < .01). Collectively, these results indicated that MEL may act through inhibition of the MAPK pathway or V‐ATPase in BC cells.

### 3.7 MEL exerts an effect on the MAPK pathway in UM‐UC‐3 and 5637 cells

**Figure 9:**
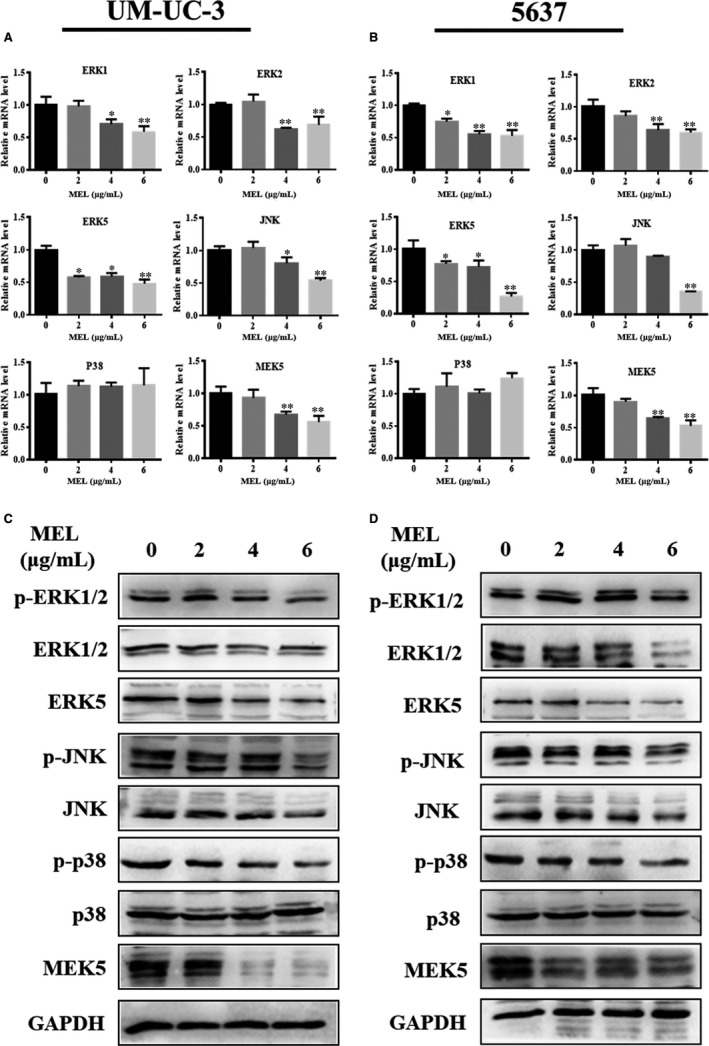
MEL exerts an effect on the MAPK pathway, as determined through qRT‐PCR and Western blotting in UM‐UC‐3 and 5637 cells. (A) The expression of star molecules of the MAPK signalling pathway (ie ERK1/2, ERK5, JNK, P38, and MEK5) was detected through qRT‐PCR in UM‐UC‐3 and (B) 5637 cells after treatment with MEL (0, 2, 4, or 6 μg/mL); *, *P* < .05; **, *P* < .01. (C) The protein and phosphorylation levels of star molecules of the MAPK signalling pathway (ie p‐ERK1/2, ERK1/2, ERK5, p‐JNK, JNK, p‐P38, P38, and MEK5) were determined through Western blotting in UM‐UC‐3 and (D) 5637 cells; *, *P* < .05; **, *P* < .01. MEL, melittin; qRT‐PCR, quantitative reverse transcription‐polymerase chain reaction

It has been reported that MEL suppresses the development and progression of tumours through inhibition of the MAPK pathway.^34^, ^35^ A number of studies outlined that the MAPK signalling pathways contain the Ras‐Raf‐ERK or classical pathway, JNK/p38 MAPK and ERK5 MAPK pathways.^36^ Studies have previously reported that MEL exerts an inhibitory effect on tumours through the classical and JNK/p38 MAPK pathways.^8^, ^34^ However, thus far, information on the role of the ERK5‐MAPK pathway in this process is limited. Therefore, we investigated the effect of MEL on the ERK5‐MAPK pathway in BC cells. Initially, the mRNA expression of star molecules (ie ERK1/2, ERK5, JNK, P38 and MEK5) of the MAPK signalling pathway was determined through qRT‐PCR in UM‐UC‐3 and 5637 cells. This analysis revealed that the expression of all genes—apart from P38—was down‐regulated in the MEL‐treated group (4 or 6 μg/mL) compared with the control group (0 μg/mL) in both cell lines (Figure 9A,B, *P* < .05, *P* < .01). Subsequently, the protein and phosphorylation levels of star molecules of the MAPK signalling pathway were analysed via Western blotting. The expression at the protein level (ERK5, MEK5, JNK and ERK1/2) and phosphorylation level (p‐ERK1/2, p‐JNK and p‐38) was evidently lower in the MEL‐treated group (4 or 6 μg/mL) compared with those observed in the control group (0 μg/mL) in both cell lines (Figure 9C,D). However, these levels were unaffected by treatment with MEL at the concentration of 2 μg/mL (Figure 9C,D). These results indicated that treatment with MEL at a certain concentration inhibits the protein and phosphorylation levels of molecules associated with the MAPK signalling pathway. Collectively, the present results demonstrate that MEL exerts an effect on the ERK5‐MAK pathway—a branch of the MAPK signalling pathway.

## 4 DISCUSSION

Reportedly, MEL possesses multifarious biological properties (ie antifungal, antiviral, antibacterial, antiparasitic and antitumour).^37^ During the previous two decades, MEL has attracted considerable attention owing to its potential use in cancer therapy.^38^, ^39^ Studies have focused on its use in the treatment of hepatocellular carcinoma,^5^, ^6^ breast cancer^7^ and lung cancer.^8^ However, its importance in BC remains undocumented.

In the present study, we identified 2015 and 4679 DEGs in normal and tumorigenic BC cells through data mining from the GEO and TCGA databases, respectively. Subsequently, GO analysis revealed that the vast majority of GEO‐identified DEGs were essentially enriched in BP terms (ie intracellular transport, protein amino acid phosphorylation and phosphate metabolic process), CC terms (ie endosome, vacuole, and membrane‐enclosed lumen) and MF terms (ie anchored to plasma membrane, zinc ion binding and enzyme binding). This finding indicated that the GEO‐identified DEGs may be involved in the progression of tumours. Subsequently, KEGG enrichment analysis demonstrated that the signalling pathways associated with these DEGs were pathways in cancer, the MAPK signalling pathway, focal adhesion, the ErbB signalling pathway and BC. Therefore, we concluded that DEGs likely encompass key molecules associated with tumour development or progression. Subsequently, according to the PPI network and MCODE analyses, we highlighted the importance of Module 2 and 4 genes in the progression of BC. Module 4 was comprised of the EPHB2, FYN, NRAS, PAK2, EPHB1, EGFR and PAK1 genes. We noted that NRAS, PAK2 EGFR and PAK1 were enriched in the MAPK signalling pathway, through which MEL may regulate tumour cells and influence their biological behaviour, as previously reported.^34^, ^35^ Module 2 was composed of the ATP6V0B, ATP6V1C1, ATP6V1E2, ATP6V0C, ATP6V1F and ATP6V0A2 genes, partly constituting the V‐ATPase domain. V‐ATPase is responsible for acidifying a variety of intracellular compartments in eukaryotic cells. Previous studies suggested that V‐ATPase may regulate a collective of BPs, such as cell growth^40^, ^41^, ^42^ and migration^43^, ^44^, ^45^ in tumours. The altered expression of V‐ATPase is considered an influential initiator and promoter of anti‐apoptosis, invasive diffusion, development of multidrug resistance and neovascularization.^46^, ^47^ Subsequently, we examined the overlap of DEGs independently identified from the GEO and TCGA databases to verify the expression of crucial module genes in BC tissues. Both original experiments were highly divergent. DEGS extracted from the GEO and TCGA poorly overlapped and only 246 DEGs in common were obtained. This may be attributed to the fact that the GEO is microarray data from human exfoliated urothelium from BC patients, while the TCGA is transcriptomic data from tumours and healthy tissue biopsies. From this overlap, we found that a majority of crucial genes in Modules 2 and 4—apart from ATP6V1E2, ATP6V0C, ATP6V0A2 and PAK1—exhibited a similar differential expression in BC tissues obtained from the TCGA database. Considering the core roles V‐ATPase and the MAPK signalling pathway play in the progression of BC, future research would investigate the effect of MEL on these crucial module genes.

Combined with the results derived from the bioinformatics analysis, the in‐vitro experiments assessed the functional role and mechanism of MEL in BC cells. Consistent with the literature,^16^ this research found that MEL inhibited cell proliferation, migration and invasion in BC cells. In fact, previous studies noted the important roles MEL plays in the progression of various cancer cells.^13^, ^14^ The importance of Modules 2 and 4 in the progression of BC was highlighted. In Module 4, the expression of NRAS, PAK2 EGFR and PAK1—enriched in the MAPK signalling pathway—was significantly decreased after treatment with MEL at a concentration of 4 or 6 μg/mL in both UM‐UC‐3 and 5673 cells. Similar findings were observed for the genes in Module 2 associated with V‐ATPase—apart from ATP6V1F. Therefore, it is possible that MEL acts through inhibition of the MAPK pathway or V‐ATPase in BC cells. It has been reported that MEL suppresses the development and progression of tumours through inhibition of the MAPK pathway, including the classical and JNK/p38 MAPK pathways.^8^, ^34^ Nevertheless, the role of the ERK5‐MAPK pathway in this process remains unknown. Subsequent investigation supported this hypothesis as follows: (a) MEL (4 or 6 μg/mL) inhibited the expression of ERK1/2, ERK5, JNK and MEK5 at the mRNA level in BC cells, as analysed using qRT‐PCR; and (b) MEL (4 or 6 μg/mL) constrained the expression of ERK5, MEK5, JNK and ERK1/2 at the protein level, as well as that of p‐ERK1/2, p‐JNK and p‐38 at the phosphorylation level. It is noteworthy that the previous research only showed that MEL affects the phosphorylation level of ERK1/2 and JNK.^48^ A possible explanation for the inhibitory effect of MEL on ERK1/2 and JNK at the protein level may be the high concentration of MEL (4 or 6 μg/mL). Although preliminary, these findings indicate that MEL exerts an effect on the ERK5‐MAK pathway—a branch of the MAPK signalling pathway.

Ultimately, this research contributed to the knowledge regarding this therapeutic setting through the identification of DEGs and crucial Module genes targeted in BC. The main finding emerging from this study is that MEL regulates the ERK5‐MAK pathway in BC cells and exerts an effect on the expressions of star molecules both at the protein and phosphorylation levels. These findings provide a theoretical basis and new insight into the clinical application of MEL. Undoubtedly, the few limitations characterizing this study cannot be ignored. This was only a preliminary investigation regarding the mechanism through which MEL influences the progression of BC cells. Further research is warranted to elucidate the mechanism through which MEL activates the MAPK signalling pathway, exerting an inhibitory effect on the progression of BC cells.

## 5 CONCLUSIONS

In summary, this research explored the effect of MEL had on the critical module genes identified in BC based on bioinformatics analysis and in vitro experiments. Our results suggested that MEL inhibited the proliferation, migration and invasion of BC cells in vitro. Moreover, our preliminary results indicated that MEL exerted an effect on a branch of the MAPK signalling pathway, namely the ERK5‐MAK pathway. This was evidenced by the inhibitory role of MEL in the expression of genes at the mRNA (ERK1/2, ERK5, JNK and MEK5), protein (ERK5, MEK5, JNK and ERK1/2) and phosphorylation (p‐ERK1/2, p‐JNK and p‐38) levels. These findings provide a theoretical basis and new insight into the clinical application of MEL.

## CONFLICT OF INTEREST

The authors confirm that there are no conflicts of interest in this work.

## AUTHOR CONTRIBUTIONS

JY conceived the study, performed the experiments and made writing‐original draft preparation. ZZ and SL provided insight and participated in data analysis. SL was responsible for data curation. ZZ and BL contributed to funding acquisition. XW helped with the writing/review of the manuscript and provided intellectual input. BL and XW contributed to supervision and project administration.

## Data Availability

The data that support the findings of this study are available from the corresponding author upon reasonable request.
