# Corrigendum

**DOI:** 10.1111/jcmm.17496

**Published:** 2022-09-05

In Yao et al,^1^ the transwell assay image of the 0 μg/mL group in Figure 6D cannot be used as a representative image. The correct figure is shown below. The authors confirm all results and conclusions of this article remain unchanged.

**FIGURE 6:**
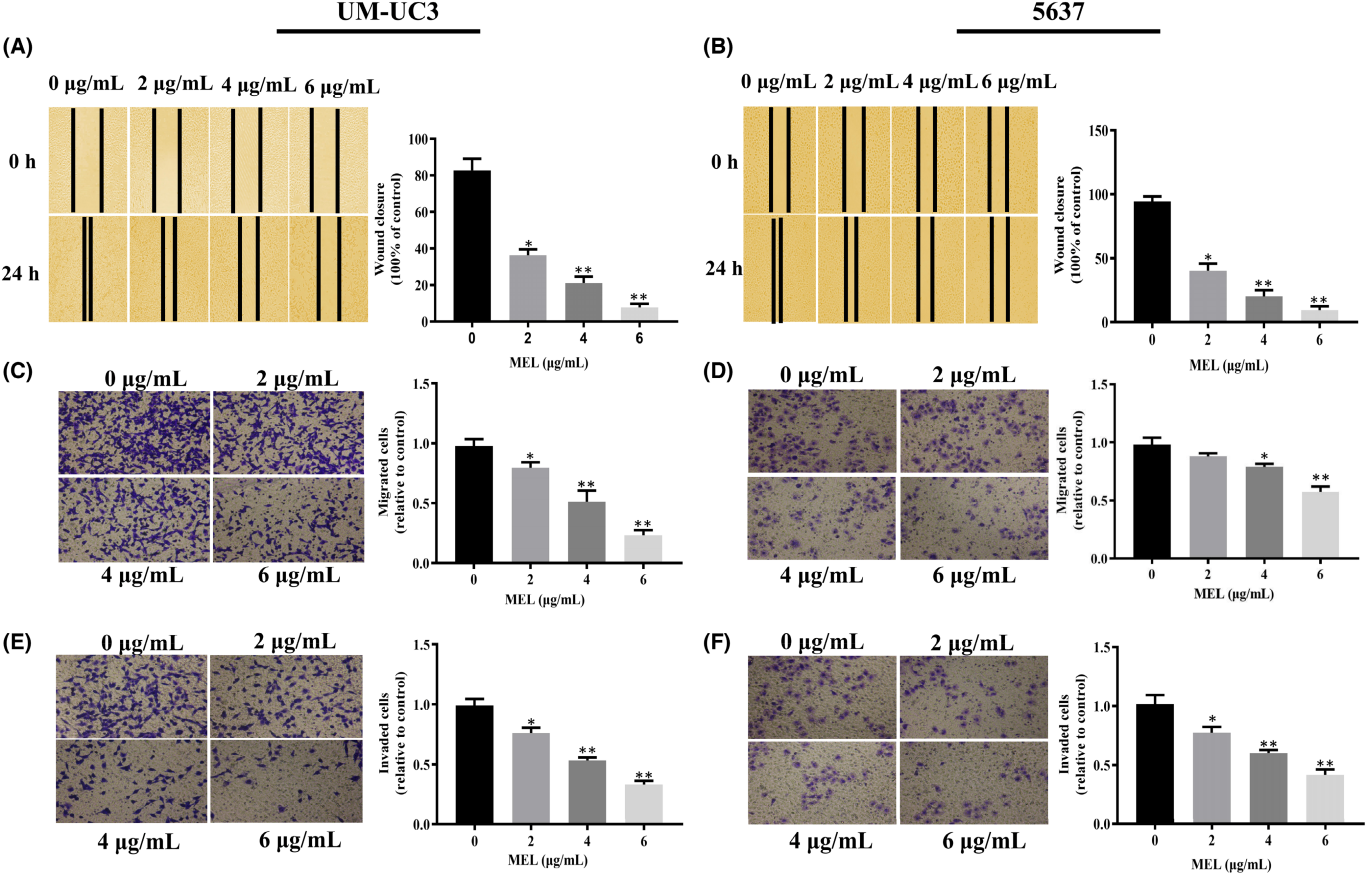
MEL‐inhibited cell migration and invasion in UM‐UC‐3 and 5637 cells. The migration of (A) UM‐UC‐3 and (B) 5637 cells was detected through the scratch wound‐healing assay after treatment with MEL (0, 2, 4 and 6 μg/mL). *, *P* < 0.05; **, *P* < 0.01. The migration of (C) UM‐UC‐3 and (D) 5637 cells was detected through the transwell assay after treatment with MEL (0, 2, 4 and 6 μg/mL). *, *P* < 0.05; **, *P* < 0.01. The invasion of (E) UM‐UC‐3 and (F) 5637 cells was detected through the transwell assay after treatment with MEL (0, 2, 4 and 6 μg/mL). *, *P* < 0.05; **, *P* < 0.01. MEL, melittin
