# Supplementary material for: Melittin inhibits proliferation, migration and invasion of bladder cancer cells by regulating key genes based on bioinformatics and experimental assays
Source: J Cell Mol Med. 2019 Nov 5;24(1):655–70. doi: 10.1111/jcmm.14775 (PMC6933335; doi:10.1111/jcmm.14775)
Supplement: Supplementary file 1 [file JCMM-24-655-s001.pdf]

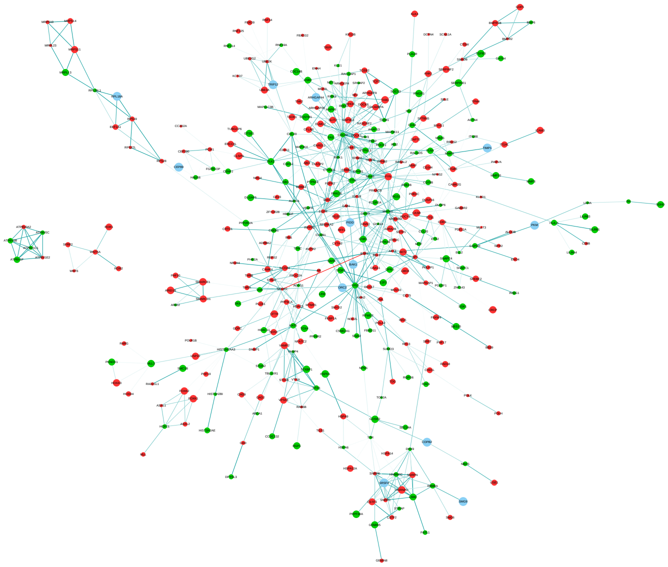

Figure S1. The PPI network constructed and visualized using the Cytoscape software (http://www.cytoscape.org/). The red nodes stand for upregulated genes, whereas the green nodes indicate downregulated genes. The blue nodes indicate genes that are not differentially expressed, but interact with DEGs in BC. The diameter of the node exhibited a negative relationship with the P value (i.e., large diameter/low P value), indicating the significance of the node. The line between two nodes shows the interaction between two genes. The depth of the line indicates the strength of their interaction. PPI, protein–protein interaction; DEGs, differentially expressed genes; BC, bladder cancer
