# Supplementary material for: Melittin inhibits proliferation, migration and invasion of bladder cancer cells by regulating key genes based on bioinformatics and experimental assays
Source: J Cell Mol Med. 2019 Nov 5;24(1):655–70. doi: 10.1111/jcmm.14775 (PMC6933335; doi:10.1111/jcmm.14775)
Supplement: Supplementary file 2 [file JCMM-24-655-s002.pdf]

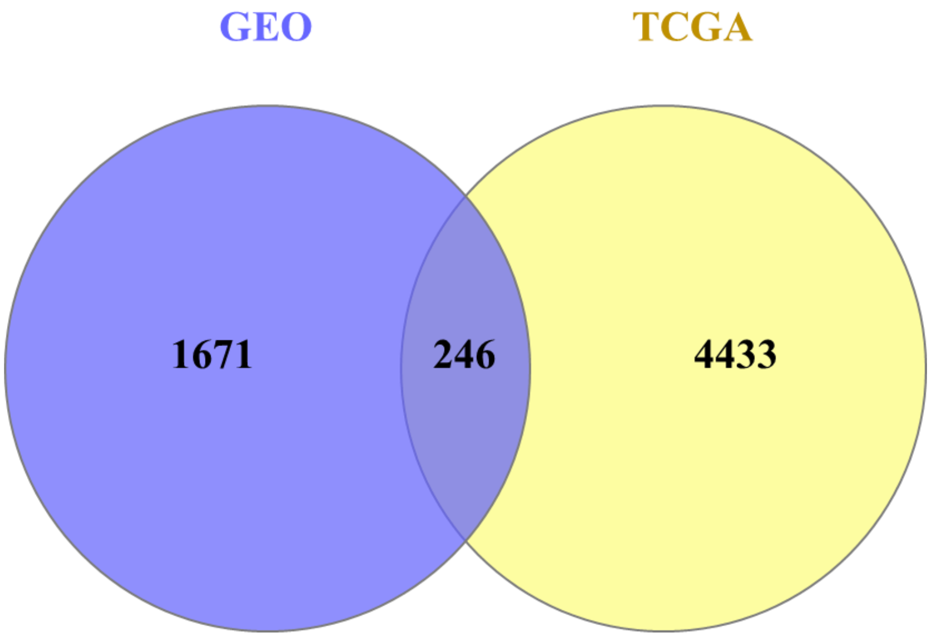

Figure S2. Venn diagram showing the overlap of genes differentially expressed in the GEO and TCGA databases. GEO, Gene Expression Omnibus; TCGA, The Cancer Genome Atlas.
