# Supplementary figures and images for: Melittin inhibits proliferation, migration and invasion of bladder cancer cells by regulating key genes based on bioinformatics and experimental assays
Source: J Cell Mol Med. 2019 Nov 5;24(1):655–70. doi: 10.1111/jcmm.14775 (PMC6933335; doi:10.1111/jcmm.14775)

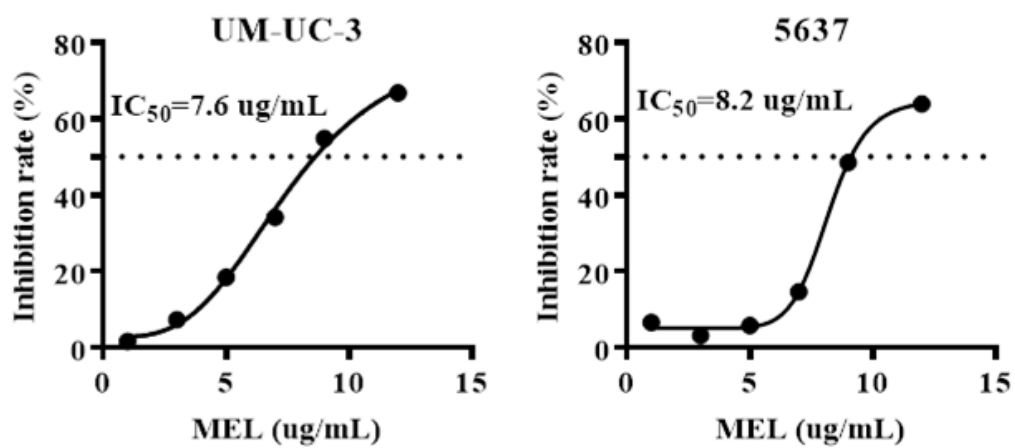

**Figure S3.** The  $IC_{50}$  of MEL was determined in UM-UC-3 and 5637 cells. MEL, melittin.

Supplement: Supplementary file 3 [file JCMM-24-655-s003.pdf]
