# Supplementary material for: Melittin inhibits proliferation, migration and invasion of bladder cancer cells by regulating key genes based on bioinformatics and experimental assays
Source: J Cell Mol Med. 2019 Nov 5;24(1):655–70. doi: 10.1111/jcmm.14775 (PMC6933335; doi:10.1111/jcmm.14775)
Supplement: Supplementary file 4 [file JCMM-24-655-s004.docx]

**Table S1.** qRT-PCR primers used in this study.

| **Genes** | **Forward primer** | **Reverse primer** |
| --- | --- | --- |
| EPHB2 | 5’-TCAAGCTCTACTGTAACGGG-3’ | 5’-GTCCCAGATGGACAACCTC-3’ |
| FYN | 5’-AAGGACTCACCGTCTTTGG-3’ | 5’-GTGTCACTCCTGTTCCTCC-3’ |
| NRAS | 5’-GATATTAACCTCTACAGGGAGC-3’ | 5’-ACACTTGTTTCCCACTAGC-3’ |
| PAK2 | 5’-CTGATCATTAACGAGATTCTGGTG-3 | 5’-TCCTACCAGGTAACTGTCCA-3’ |
| EPHB1 | 5’-GCTATGGGATCGTCATGTG-3’ | 5’-CATTGATGACATCTTGGTTGG-3’ |
| EGFR | 5’-TCCTGGAGAAAGGAGAACG-3’ | 5’ATCATCCAGCACTTGACCA-3’ |
| PAK1 | 5’-TTCCGGGACTTTCTGAACC-3’ | 5’-GAATTGATGCTGTAGCAGCTC-3’ |
| ATP6V0B | 5’-CTATATTACCGGCTCCTCCA-3’ | 5’-AAGATGATGCTGACCAGGT-3’ |
| ATP6V1C1 | 5’-AGGTCTAGCAATGTTCTTTCAG-3’ | 5’-GAAGTCATCAACTGCCTTCC-3’ |
| ATP6V1E2 | 5’-CATCTTCTTAGAAGTCACTGAACC-3’ | 5’-CATCCACTATGGAAGAACAACTC-3’ |
| ATP6V0C | 5’-ATCATCCCAGTGGTCATGG-3’ | 5’-AGGAAGCTCTTGTAGAGGC-3’ |
| ATP6V1F | 5’-CACTTTCCGTTCACTTGGA-3’ | 5’-ATTTCATCCCAAAGCGGAG-3’ |
| ATP6V0A2 | 5’-GGATCTCTACACTGTACTGCA-3’ | 5’-CACGGCTGTAGACAGACTC-3’ |
| ERK5 | 5’-AAATCTGTCTACGTGGTCCTG -3’ | 5’-TTGGTACAGGAAGTAGCGC-3’ |
| MEK5 | 5’-TTGTTGATGAGGATTCGCC-3’ | 5’-TCAATTCTTCAGGTGCTGG-3’ |
| ERK1 | 5’-ATCTAAAGCCCTCCAACCTG-3’ | 5’-CATACTCCGTCAGGAAGCCG-3’ |
| ERK2 | 5’-GATCTCAAGATCTGTGACTTTGG-3’ | 5’-CACATATTCTGTCAGGAACCC-3’ |
| JNK | 5’-ACTTAAAGCCAGTCAGGCA-3’ | 5’-GAGCTTCATCTACAGAGATCCT-3’ |
| p38 | 5’-GAACAAGACAATCTGGGAGG-3’ | 5’-TGAATGATGGACTGAAATGG-3’ |
| β-Actin | 5’-GTGCCCATCTACGAGGGGTATG-3’ | 5’-TGAGGTAGTCAGTCAGGTCCCG-3’ |

Abbreviations: qRT-PCR, quantitative real-time polymerase chain reaction.
